# Gold nanoparticles covalently assembled onto vesicle structures as possible biosensing platform

**DOI:** 10.3762/bjnano.7.58

**Published:** 2016-05-02

**Authors:** M Fátima Barroso, M Alejandra Luna, Juan S Flores Tabares, Cristina Delerue-Matos, N Mariano Correa, Fernando Moyano, Patricia G Molina

**Affiliations:** 1 Departamento de Química. Facultad de Ciencias Exactas, Físico-Químicas y Naturales. Universidad Nacional de Río Cuarto. Agencia Postal Nº 3 - (5800) Río Cuarto, Argentina; 2 REQUIMTE/LAQV, Instituto Superior de Engenharia do Porto, Instituto Politécnico do Porto, Rua Dr. António Bernardino de Almeida 431, 4200-072 Porto, Portugal

**Keywords:** covalently decorated vesicles, gold nanoparticles, immunosensors design, synthesis

## Abstract

In this contribution a strategy is shown to covalently immobilize gold nanoparticles (AuNPs) onto vesicle bilayers with the aim of using this nanomaterial as platform for the future design of immunosensors. A novel methodology for the self-assembly of AuNPs onto large unilamellar vesicle structures is described. The vesicles were formed with 1,2-dioleoyl-*sn*-glycero-3-phosphocholine (DOPC) and 1-undecanethiol (SH). After, the AuNPs photochemically synthesized in pure glycerol were mixed and anchored onto SH–DOPC vesicles. The data provided by voltammetry, spectrometry and microscopy techniques indicated that the AuNPs were successfully covalently anchored onto the vesicle bilayer and decorated vesicles exhibit a spherical shape with a size of 190 ± 10 nm. The developed procedure is easy, rapid and reproducible to start designing a possible immunosensor by using environmentally friendly procedures.

## Introduction

Vesicles are spherical structures composed primarily of phospholipids and these are organized in bilayers. These vesicles contain an internal aqueous phase and are suspended in an external aqueous phase. Therefore, the vesicles may contain lipophilic substances, which are located in the hydrophobic bilayer as well as water-soluble substances. These structures are the best mimetic agents of biological membranes and represent the environment in which many proteins and enzymes show activity [1–2]. The advantages that the vesicles have over synthetic materials are: lack of toxicity, biodegradability and biocompatibility, so that they are utilized as versatile carriers in the fields of medical and analytical applications [1–4]. Several strategies employing vesicles in analytical fields have been reported, namely their use as signal amplifiers in the development of biosensors [3,5] to fulfil performance criteria such as high sensitivity and low limit of detection. In this context, vesicles can be used in the construction of biosensors as supporting film to coat Au or Ag electrodes [3]. Self-assembly of nanoparticles onto organised systems combines the advantages of nanomaterials – very small size, high loading of signal tags, high surface area and dynamic character for signal amplification [6].

Some types of nanomaterials have been self-assembled into vesicle structures for different purposes. Béalle et al. [7] describe the use of super-paramagnetic iron oxide nanoparticles to decorate vesicles that could be used as a model system to illustrate controlled delivery of molecules under mild hyperthermia. These systems were prepared by using cetyltrimethylammonium chloride and myristic acid, and the nanomaterial was synthetized in aqueous alkaline solution by a co-precipitation process of FeCl_2_ and FeCl_3_. Using an adsorptive process the vesicles structure was incorporated with super-paramagnetic iron oxide nanoparticles [7]. Additionally, the molecular deposition of silica from water was carried out [8]. Silica-coated unilamellar surfactant vesicles were prepared by hydrolysis and condensation of silicon alkoxides into organized inorganic materials (dioctadecyldimethylammonium bromide and didodecyldimethylammonium bromide).

Thus, gold nanoparticles (AuNPs) form stable complexes with hydrophobic drugs and dyes. These drugs and dyes are successfully released into cells [9]. The AuNPs were prepared in aqueous media, with a diameter of 2.5 nm, and were used to decorate amphiphilic monolayers composed by a hydrophobic alkanethiol and hydrophilic tetraethylene glycol. Furthermore, it was reported the usage of AuNPs of 27 nm of diameter to decorate small receptors composed by per-6-thio-β-cyclodextrin accomplished via covalent gold–thiol bonds [10]. These AuNPs provide an excellent platform for drug delivery due to the functional versatility of their monolayers. The vesicles with AuNPs are suitable for applications such as transport inside cells [11], photodynamic inactivation and also in biosensors [12].

There are many properties that depend on the shape of the nanostructures. In this regard, there have been many studies with different ways to synthesize them [13–17]. For example, tips and edges located in the nanoparticles have regions of high electric fields, which improve the optical effects [16]. Also, nanoparticles with different faces having different densities of adsorption sites and may exhibit different catalytic properties [18].

Some reports [19–20] have demonstrated that metallic nanoparticles (gold and silver) can slowly precipitate in vesicle systems and, in doing so, lose the specific qualities of nanoparticles [21]. Moreover, the conventional synthesis of AuNPs is carried out in water and uses chemicals such as NaBH_4_, which is toxic and can damage functional groups. In addition, some nanomaterial syntheses need involve high temperatures, which destroy or modify these systems [11].

**Figure 1:**
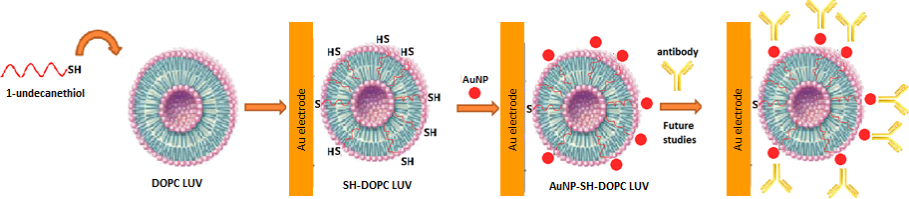
Platform to design a possible immunosensor.

In this contribution a strategy is shown to covalently immobilize AuNPs onto vesicle bilayers with the aim of using this nanomaterial as a platform for the future design of immunosensors (Figure 1), which can be used to detect different analytes by electrochemical impedance spectroscopy or square wave voltammetry [22–23]. It is known that antibodies can be immobilized onto AuNPs without losing their biological properties [24–25]. Thus, the covalent immobilization of vesicles decorated with AuNPs on a gold surface could increase the amount of immobilized antibodies, which would result in an increase in sensitivity. Moreover, since the conjugate antigen–antibody is broken with changes of the values of pH or ionic strength [25], is very important that the AuNPs are covalently attached to the vesicles. Otherwise, if the binding is electrostatic, the AuNPs will be lost. In this way only the antigen–antibody complex would be broken and the sensor will be reusable.

Although, the preparation of nanomaterials in aqueous and organic media has been extensively studied, to our knowledge, there are no studies concerning the surface decoration of vesicles by using AuNPs photochemically synthetized in glycerol and using a covalent bond between the nanoparticle and the surface. Recently, we have reported [26] the synthesis of AuNPs in pure glycerol without additional stabilizers and using ultraviolet irradiation for a few minutes. In this synthesis, the glycerol acts as the solvent for the synthesis reaction as well as the reducing agent.

Therefore, this work describes the self-assembly of AuNPs photochemically synthesized with two different irradiation times (8 and 13 min) in glycerol onto vesicle structures. After the optimization of some analytical features of the AuNPs synthesis (irradiation time, HAuCl_4_ concentration), these AuNPs were covalently immobilized onto large unilamellar vesicles (LUVs) composed by the phospholipid DOPC (1,2-dioleoyl-*sn*-glycero-3-phosphocholine). For this purpose 1-undecanethiol (SH) was incorporated into the bilayer in order to covalently bind the AuNPs to the bilayer and to immobilize the vesicle on the Au electrode. This nanosystem was characterized by using UV–vis spectroscopy, transmission electron microscopy (TEM), dynamic light scattering (DLS) and cyclic voltammetry (CV). The results show that the vesicles were decorated with AuNPs with the advantage that the method is fast, non-polluting and reliable since no nanoparticles extraction is needed. This study offers a basic understanding of a new nanomaterial and its characteristics can help future applications.

## Results and Discussion

This work reports different studies performed on the surface decoration of vesicle structures with AuNPs formed in pure glycerol by using a photochemical process with two irradiation times (8 and 13 min). For that, firstly the AuNP synthesis and the vesicles preparation were carried out. After that the self-assembly of the AuNPs onto modified vesicle structures (SH–DOPC LUVs) was promoted (see Figure 1). Several techniques were used to characterize this new system, which is described below.

### Characterization of AuNPs

Usually, the synthesis of AuNPs is performed in aqueous media by promoting a chemical reaction between a gold precursor and a reducing agent [27–28]. After this chemical process, it is necessary to extract the AuNPs from the reaction media in order to use them in the desired scientific field. Recently, we reported [26] an alternative and simple approach to synthesize AuNPs in an organic medium, namely glycerol, by using a photochemical process instead of a chemical process. Under our experimental conditions, we observed that 8 min is enough to convert Au^3+^ ions in Au^0^ nanoparticles in glycerol (the typical change of colour from yellow to pink appears). We have seen as a novelty that the nanoparticles are very sensible to the conditions under which they are formed. As the irradiation time modifies the nanoparticle morphology we decided to irradiate for longer time, in this case, 13 min. Some techniques, such as UV–vis spectroscopy and TEM were used to perform the morphological characterization of the AuNPs.

**Figure 2:**
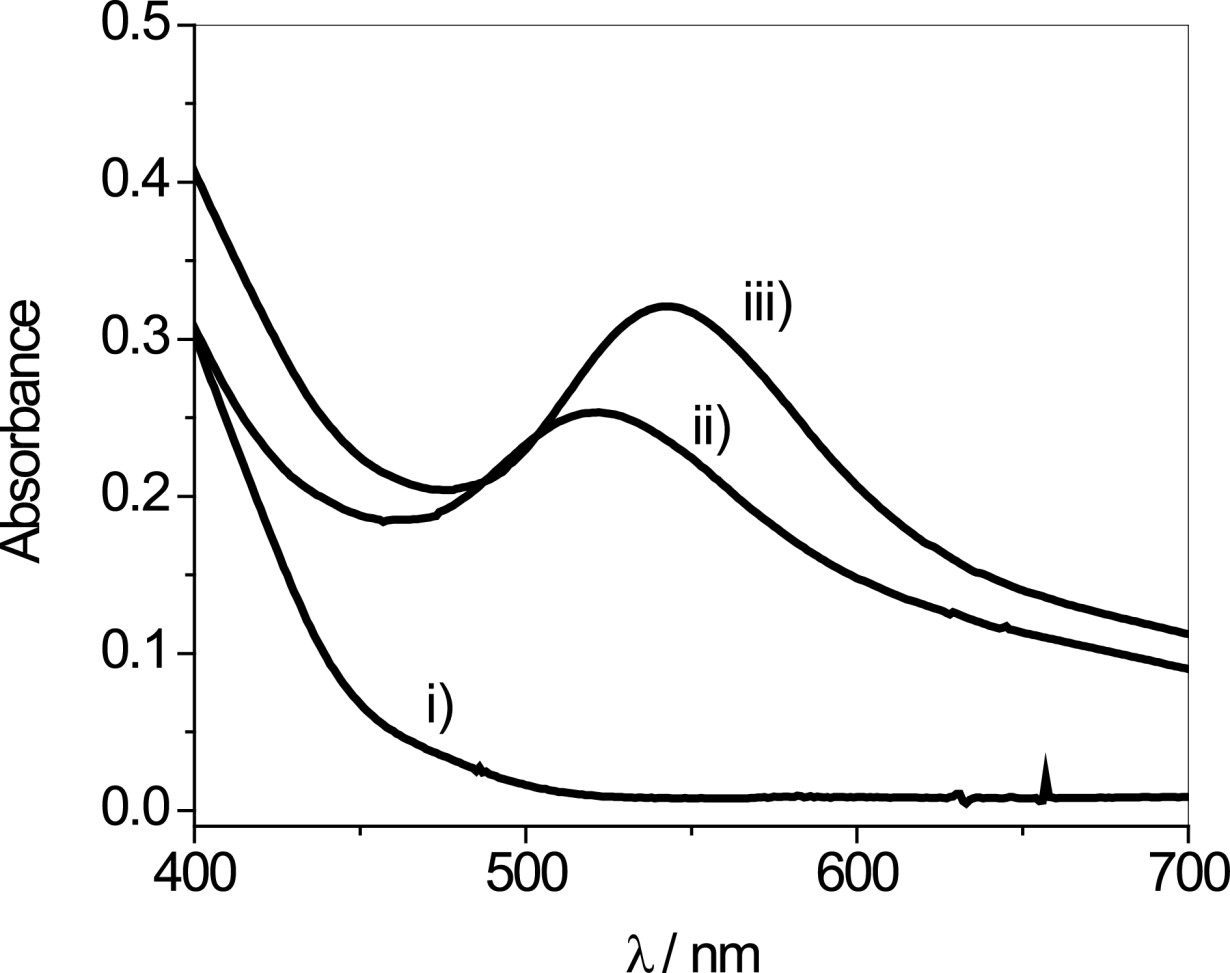
UV–vis spectra in glycerol: i) the HClAu_4_ precursor; ii) the AuNPs synthesized with 8 min of irradiation time; iii) the AuNPs synthesized with 13 min of irradiation time (λ_irradiation_ = 300 nm).

Figure 2 shows the absorption spectra of the glycerol solutions of the metallic precursor and the AuNPs synthesized with different times of irradiation (λ_irridiation_ = 300 nm). Glycerol acts as solvent and as the reducing agent and the glycerol oxidation products are able to stabilize the AuNPs [26]. Note, that when the AuNPs are formed after 8 and 13 min of UV irradiation, bands corresponding to the surface plasmon resonance (SPR) around 520 or 550 nm appeared. This maximum wavelength (λ_max_) is in accordance with other reported studies [26,29–30], and the small differences founded in the λ_max_ may indicate different sizes of the obtained nanoparticles. Besides, the UV–vis spectra exhibit a band half-width of around 50 nm suggesting a narrow size of distribution of AuNPs. We highlight that the AuNPs solutions were prepared without any additional stabilizer and that they remain stable for two months. After this period, λ_max_ shifts to the blue (hypsochromic shift) and the solutions turn blue, indicating that the AuNPs are aggregated.

**Figure 3:**
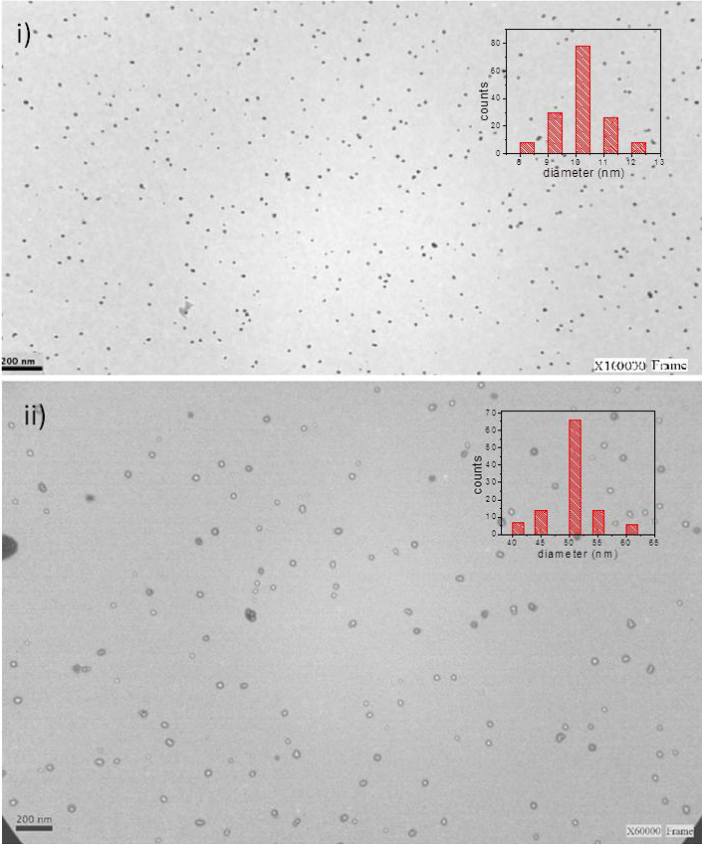
TEM images of AuNPs synthesized in glycerol by using ultraviolet irradiation for i) 8 min and ii) 13 min. The inset gives the histograms of diameters of the AuNPs based on the inspection of i) 150 particles and ii) 110 particles.

In order to show the morphology of the AuNPs, TEM images of different nanoparticles are shown in Figure 3. As it can be observed, the AuNPs formed after 8 min of UV radiation are shape with a size of 10 ± 2 nm (Figure 3i), while the AuNPs formed after 13 min exhibit a hollow spherical shape with a size of 50 ± 10 nm (Figure 3ii). Recently, the hollow nanoparticles have been used in the construction of biosensors because they can accelerate the transfer of electrons due to their excellent electrocatalytic activity [17]. For this reason, studies were conducted to immobilize both types of nanoparticles on the vesicles as will be shown below.

### Characterization of vesicles

#### Effect of glycerol on the stability of the vesicles

Considering that the nanoparticles synthesized in glycerol are not extracted from the reaction medium and will be subsequently immobilized on SH–DOPC LUVs, these LUVs were prepared with different percentage of glycerol in order to verify if this organic solvent has some effect on their size measured by DLS.

**Figure 4:**
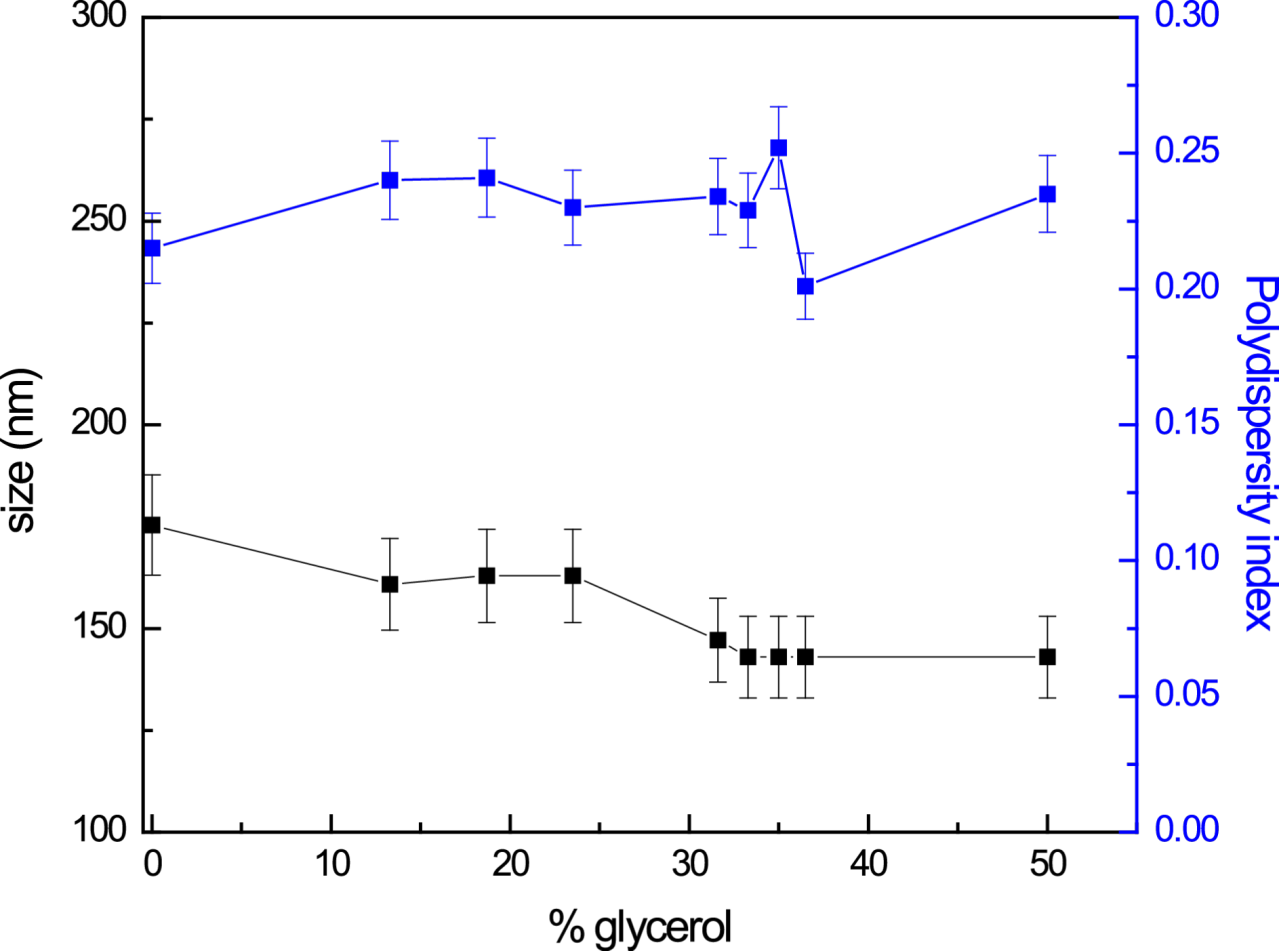
Variation of diameter and polydispersity index of SH–DOPC LUVs with glycerol content.

Figure 4 shows the effect that glycerol content, ranging from 0 to 50% (v/v), has on the diameter and on the polydispersity index of the SH–DOPC LUVs. The sizes of the SH–DOPC LUVs mixed with glycerol were between 145 and 175 nm and the polydispersity index was between 0.20 and 0.25. Therefore, the glycerol has no influence on the size and the polydispersity index of the SH–DOPC LUVs since these values are within the experimental error.

#### Optimization of the SH/DOPC ratio for electrochemical measurements

Different SH/DOPC ratios in the formation of vesicles were explored in order to find the optimal conditions for anchoring the AuNPs on the vesicles. The LUV solutions were prepared in the same phosphate buffer solution, which is used for electrochemical measurements, employing different molar ratios of SH and DOPC as it is described in subsection “Vesicles preparation” of the Experimental section. All diameters of these organized systems were around 175 nm with a polidispersity of 0.2 determined by DLS indicating that the size of the LUVs does not change significantly by the incorporation of SH to the bilayer.

A question may arise here about the orientation of the thiol molecules in the bilayer. That is, whether there are thiol molecules anchored in the bilayer oriented to the outer water phase. CV was used in order to determine the incorporation and orientation of SH in the bilayer of DOPC LUVs since, the SH molecules have the function of immobilizing the AuNPs onto the bilayer and to anchor the decorated vesicles on the Au electrode (AuE). For that, a simple and well-defined redox system was used consisting of K_4_[Fe(CN)_6_] [26], which first was employed to characterize the surface properties of the AuE modified with the SH–DOPC LUVs without AuNPs.

**Figure 5:**
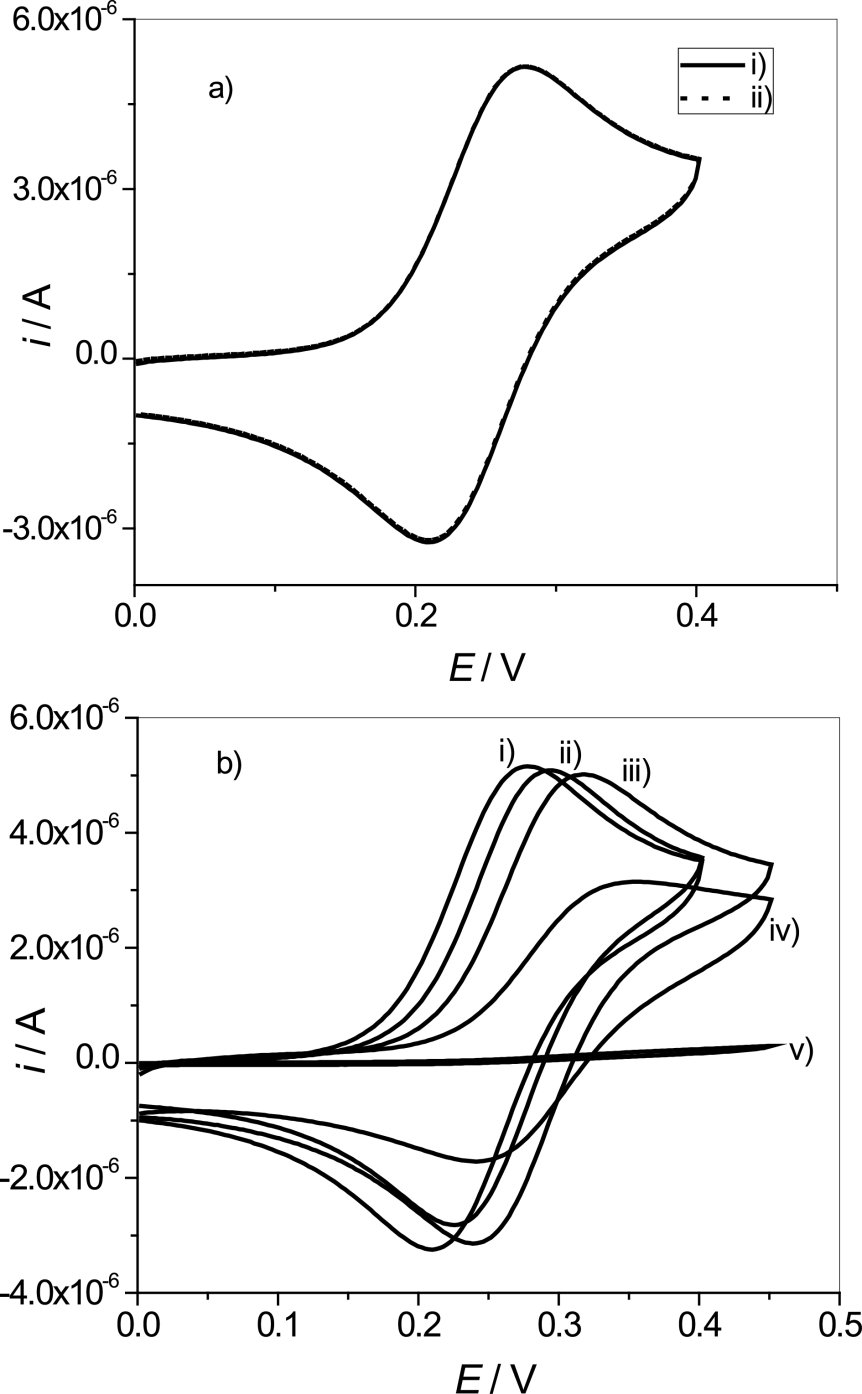
Cyclic voltammograms of K_4_[Fe(CN)_6_] in phosphate buffer solution (pH 7.0) using a) (i) bare AuE , (ii) AuE previously immersed in a solution of DOPC LUVs for 30 min, washed and taken to the electrochemical cell. b) Cyclic voltammograms of K_4_Fe(CN)_6_ in phosphate buffer solution (pH 7.0) by using AuE previously immersed in different SH–DOPC LUV solutions for 30 min, washed and taken to the electrochemical cell. The following SH/DOPC molar ratios were used: i) bare AuE ii) 0.003:1; iii) 0.033:1; iv) 0.33:1 and v) 0.66:1 (
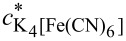
 = 1 × 10^−3^ M; *v* = 100 mV·s^−1^).

To perform this electrochemical characterization, firstly a clean AuE surface was exposed and immersed in the SH–DOPC LUV solution for 30 min, enough time to form bonds between AuE and SH/DOPC [25]. This procedure was carried out in order to covalently bond the vesicles on the AuE through the thiol incorporated in the bilayer. Then, the AuE modified with SH–DOPC LUVs was immersed in an aqueous solution of 1.0 × 10^−3^ M [Fe(CN)_6_]^4−^ in phosphate buffer solution (pH 7.0) and CV was carried out in the potential range of 0.0–0.4 V vs AgCl/Ag. Figure 5 shows the cyclic voltammograms obtained in these experiments. In Figure 5a a reversible redox peak of [Fe(CN)_6_]^4−^ at +0.277 V (anodic potential peak) and +0.210 V (cathodic potential peak) vs AgCl/Ag was observed when a bare AuE or a AuE previously immersed for 30 min in a solution with DOPC LUVs (without the thiol incorporation) was used. This indicates that DOPC LUVs are not adsorbed on the AuE surface. However, when the AuE surface was modified with LUVs formed at different SH/DOPC ratios, a decrease of the peak intensity of [Fe(CN)_6_]^4−^ and a displacement of the peak potentials (oxidation and reduction) was observed (Figure 5b). This behaviour can be explained by invoking the resistance to the electron transport generated by the SH–DOPC LUVs covalently bonded to the AuE surface (Figure 1).

As the SH/DOPC ratio was increased, a decrease of the [Fe(CN)_6_]^4−^ peak intensity of the current (oxidation and reduction) was observed. Moreover, at a ratio of SH/DOPC = 0.66:1, the [Fe(CN)_6_]^4−^ couple redox peak intensity of the current decreases drastically indicating that the AuE surface was blocked and the electron transfer between the modified electrode surface and the [Fe(CN)_6_]^4−^ is hindered.

**Figure 6:**
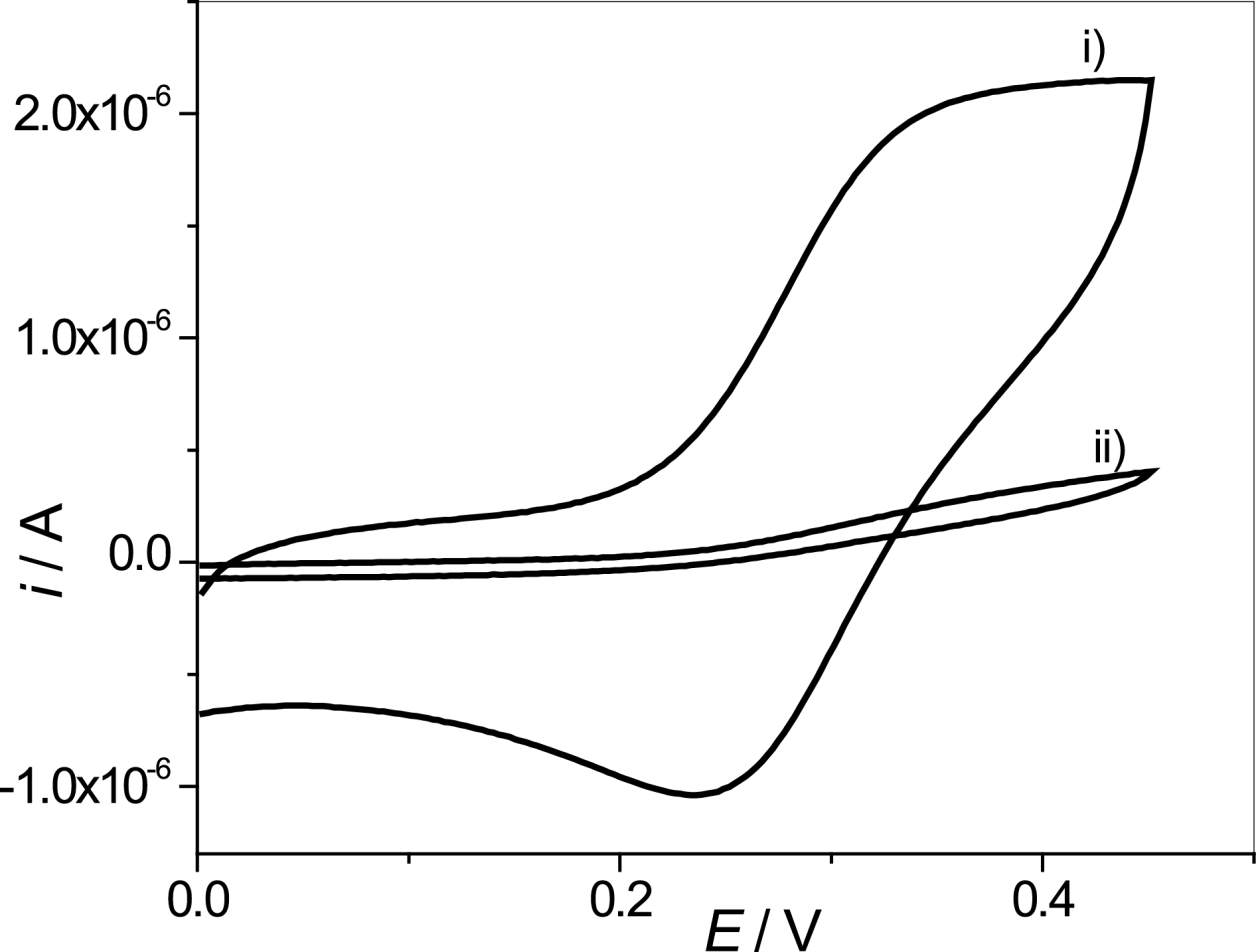
Cyclic voltammograms of K_4_[Fe(CN)_6_] generated in phosphate buffer solution (pH 7.0) by using AuE previously modified with AuNPs–SH–DOPC LUVs using SH/DOPC molar ratios of i) 0.33:1 and ii) 0.66:1 (
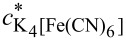
 = 1 × 10^−3^ M, *v* = 100 mV·s^−1^).

The next step was to verify the electrochemical behaviour with regard to [Fe(CN)_6_]^4−^ of the decorated vesicles (AuNPs–SH–DOPC LUVs) with AuNPs formed after 8 min anchored on AuE, by using SH/DOPC ratios of 0.33:1 and 0.66:1. Figure 6 shows the electrochemical studies of an electrode modified with AuNPs–SH–DOPC LUVs for both ratios of SH/DOPC. At an SH/DOPC ratio of 0.33:1 a good and reproducible electrochemical signal was obtained (Figure 6i), whereas for the ratio of 0.66:1 the current falls dramatically (Figure 6ii). This means that the 0.66:1 SH:DOPC ratio has limitations considering the use as a platform for an electrochemical immunosensor, since in its development it is required to obtain an electrochemical response that scales with the antigen concentration.

It should be noted that, the same procedure was performed using modified electrodes with decorated vesicles with AuNPs formed after 13 min but no reproducible voltammograms were obtained (data not shown).

#### TEM measurements of AuNPs–SH–DOPC LUVs

**Figure 7:**
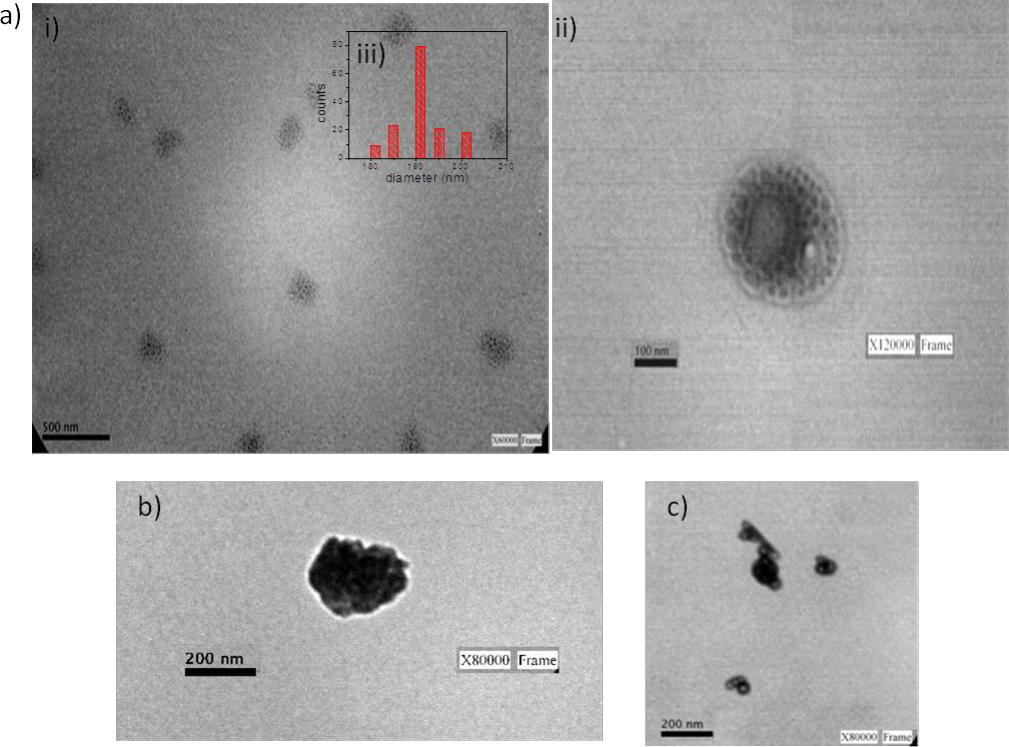
TEM images of AuNPs–SH–DOPC LUVs. a) i) and ii) TEM images of AuNPs–SH–DOPC LUVs using AuNPs synthesized in glycerol with 8 min of irradiation time and LUVs prepared with a SH/DOPC molar ratio of 0.33:1. iii) Diameter histograms of the AuNPs–SH–DOPC LUVs based on the inspection of 150 LUVs. b) AuNPs–SH–DOPC LUVs using AuNPs synthesized in glycerol with 8 min of irradiation time and LUVs prepared with a SH/DOPC molar ratio of 0.66:1. c) AuNPs–SH–DOPC LUVs by using AuNPs synthesized in glycerol with 13 min of irradiation time and LUVs prepared with a SH/DOPC molar ratio of 0.33:1.

In order to obtain the size and the morphology of the AuNPs–SH–DOPC LUV systems, TEM images were recorded. Figure 7a,b shows the TEM images of the AuNPs–SH–DOPC LUVs by using AuNPs formed after 8 min for SH/DOPC ratios of 0.33:1 and 0.66:1, respectively. As can be seen, the AuNPs were successfully anchored on the vesicle structures. Furthermore, the AuNPs anchored on the SH–DOPC LUVs with a molar ratio of 0.33:1 (Figure 7a) present a well-defined morphology with spherical shape and a size of 190 ± 10 nm. Figure 7b shows that surface of the vesicles becomes saturated with AuNPs when a 0.66:1 SH/DOPC ratio is used. This could affect the anchoring of the antibodies in futures studies because a certain spacing of the AuNPs is required for its proper orientation [25]. Figure 7c shows a TEM image of the LUVs decorated with AuNPs formed after 13 min of irradiation. The morphology of the AuNPs anchored on the vesicles shows hollow, nearly spherical structures as it was shown in Figure 3ii but aggregations without defined structures are observed. Hence, the major part of the hollow AuNPs assembles in a non-uniform manner on the vesicles, and the reproducibility of the sensor would be affected.

Based on these results, using AuNPs formed after 8 min of irradiation and a molar SH/DOPC ratio of 0.33:1 yields the following advantages: a) decorated vesicles of regular size are formed, which would favour the reproducibility of the sensor b) AuNPs are conveniently spaced to anchor the antibody correctly and c) – as discussed previously in subsection “Optimization of the SH/DOPC ratio for electrochemical measurements“ – the electrochemical signal on the electrode opens the possibility for a future design of an immunosensor.

## Conclusion

In the present work, the covalent self-assembly of AuNPs on vesicles with thiol incorporated in the bilayer was achieved. The developed procedure consisted of mixing AuNPs photochemically synthesized in glycerol media in vesicle structures composed by DOPC and 1-undecanethiol with the different molar ratios. The developed methodology is rapid and easy to perform and provides a non-polluting and sustainable methodology. This nanomaterial offers interesting possibilities and future applications such as its use in the design of immunosensors. Moreover, the developed methodology can be a promising candidate for other applications as optical addressable delivery system and affinity columns.

## Experimental

### Synthesis of gold nanoparticles

AuNPs were synthesized by using a solution of 1.15 × 10^−3^ M HAuCl_4_ (SIGMA, USA) in glycerol (Sintorgan, HPLC grade). Briefly, the solution of HAuCl_4_ in glycerol was placed in a 2 mL quartz cuvette and exposed to ultraviolet irradiation (300 nm) for 8 or 13 min. The Rayo Net chamber lamps (Model RPR-100) yield 32 W and controlled by dual switches located on the front panel. The dimensions of the reactor chamber were 16 inch depth and 10 inch diameter, with 2/8 inch openings at the top [26]. The AuNPs solutions were of pink color and were stored in a dark glass bottle at 6 °C for further use.

### Vesicles preparation

The large unilamellar vesicles (LUVs) were formed, at 20.0 ± 0.1 °C, by using the phospholipid 1,2-di-oleoyl-*sn*-glycero-3-phosphatidylcholine (DOPC) which has a phase transition temperature of −17.3 °C [31]. The DOPC solution in chloroform was obtained from Avanti Polar Lipids, Inc., USA. The LUVs of DOPC loaded with 1-undecanethiol (SH) were prepared by the extrusion method [32]. The typical procedure was: a) An appropriate amount of DOPC was transferred into a volumetric flask by using a calibrated microsyringe and then SH solution in chloroform is added to reach the desired concentration (Sigma, USA). Always, the final concentration of DOPC was 1.27 × 10^−3^ M and the SH/DOPC ratios prepared were 0.0033:1; 0.033:1; 0.33:1 and 0.66:1. b) The chloroform was evaporated and the film was dried under reduced pressure. c) Large multilamellar vesicles (LMVs) of SH–DOPC were obtained by hydrating the dry lipid film through mixing (vortex-2-Genie) for about 5 min at room temperature with using ultrapure water (Labconco equipment model 90901-01) or phosphate buffer solution (pH 7.0, Merck) to carry out the morphologic/topographic or the electrochemical studies, respectively.

During the preparation of LUVs the following procedure was followed [1]: The LMV suspension was extruded ten times (Extruder, Lipex biomembranes) through two stacked polycarbonate filters of pore size 200 nm under nitrogen pressure up to 3.4 atm. In order to obtain the decorated vesicles (AuNPs–SH–DOPC LUVs) 5 mL of a solution of SH–DOPC LUVs and 1 mL of different AuNPs glycerol solutions (irradiation times of 8 and 13 min) are mixed and they are allowed to react for 30 min. All samples were used immediately after preparation and the presence of decorated AuNPs–SH–DOPC LUV was verified by different techniques discussed below.

### Nanomaterial characterization

#### Dynamic light scattering (DLS)

The diameters of the LUVs were determined by DLS (Malvern 4700 with goniometer and 7132 correlator) with an argon ion laser operating at 488 nm. All the measurements were performed by using a scattering angle of 90° at a temperature of 20.0 ± 0.1 °C. The measurements were made by diluting the samples in a cuvette with distilled water. The water was filtered three times by using an Acrodisc with 0.45 µm Nylon membrane (Agilen) to remove dust or particles. To obtain valid results from DLS measurements, the knowledge of the refractive index and the viscosity of the system is required in addition to well-defined conditions [33]. Since we worked with diluted solutions, the refractive indices and viscosities for the vesicle solutions were assumed to be the same as those of the external solvent [34]. Multiple samples for each size were made, and thirty independent size measurements were made for each individual sample at the scattering angle of 90°. The instrument was calibrated before and during the course of experiments by using several different size standards. Thus, we are confident that the magnitudes obtained by DLS measurements can be statistically significant for all the systems investigated. The algorithm used was CONTIN and the DLS experiments shown that the polydispersities of the LUVs were less than 5%.

#### UV–vis spectroscopy

In order to study the formation of AuNPs, UV–vis studies were performed by using Shimadzu 2401 equipment. The path length used in the absorption measurement was 1 cm.

#### Transmission electron microscopy (TEM)

The observation of the TEM micrographs was performed by using a Philips CM-12 microscope at 20–120 kV with a Megaview-II Docu camera and SIS NT Docu software. To carry out the experiment a drop of AuNPs or AuNPs–SH–DOPC LUV was suspended onto a copper coated grid and dried in a desiccator.

#### Cyclic voltammetry (CV)

CV was performed with an Autolab PGSTAT 10 potentiostat controlled by GPES software (EcoChemie, Netherlands). A conventional three-electrode cell was used, which included a Au electrode (0.07 cm^2^) as the working electrode, a Pt counter electrode and a Ag|AgCl|KCl_sat_ reference electrode. Before used, the Au electrode was polished, sonicated and rinsed with ultrapure water. CV measurements were carried out in aqueous media by using as molecular probe K_4_[Fe(CN)_6_] (1 × 10^−3^ M) in phosphate buffer solution (pH 7.0) at a scan rate of 100 mV·s^−1^ and using a potential scan between +0.0 and +0.4 V vs AgCl/Ag. For the electrochemical studies, the Au electrode was placed in different DOPC LUV and SH–DOPC LUV solutions for 30 min. Then the Au electrode was rinsed and placed into the cell containing K_4_[Fe(CN)_6_] (1 × 10^−3^ M) in a phosphate buffer solution (pH 7.0) to record the voltammograms. For the electrochemical experiments with the decorated vesicles first SH–DOPC LUV were immobilized on the Au electrode as described above, then the modified Au electrode was immersed in the glycerol solution containing the AuNPs for 30 min. The modified Au electrode was subsequently rinsed and taken to the electrochemical cell.
